# Development and validation of a prediction model for tuberculous pleural effusion: a large cohort study and external validation

**DOI:** 10.1186/s12931-022-02051-4

**Published:** 2022-05-27

**Authors:** Yanqing Liu, Zhigang Liang, Songbo Yuan, Shanshan Wang, Fei Guo, Weidong Peng, Jing Yang, Aihua Wu

**Affiliations:** 1 Department of Laboratory Medicine, Ningbo First Hospital, 59 Liuting Street, Ningbo, 315010 Zhejiang China; 2 Department of Thoracic Surgery, Ningbo First Hospital, Ningbo, Zhejiang China; 3 Department of Clinical Laboratory, The Affiliated People Hospital of Ningbo University, Ningbo, Zhejiang China; 4 Department of Respiratory and Critical Care Medicine, The Affiliated People Hospital of Ningbo University, Ningbo, Zhejiang China; 5 Department of Respiratory and Critical Care Medicine, Ningbo First Hospital, Ningbo, Zhejiang China

**Keywords:** Tuberculous pleural effusion, Nomogram, Scoring system, Adenosine deaminase, Area under the curve

## Abstract

**Background:**

Distinguishing tuberculous pleural effusion (TPE) from non-tuberculosis (TB) benign pleural effusion (BPE) remains to be a challenge in clinical practice. The aim of the present study was to develop and validate a novel nomogram for diagnosing TPE.

**Methods:**

In this retrospective analysis, a total of 909 consecutive patients with TPE and non-TB BPE from Ningbo First Hospital were divided into the training set and the internal validation set at a ratio of 7:3, respectively. The clinical and laboratory features were collected and analyzed by logistic regression analysis. A diagnostic model incorporating selected variables was developed and was externally validated in a cohort of 110 patients from another hospital.

**Results:**

Six variables including age, effusion lymphocyte, effusion adenosine deaminase (ADA), effusion lactatedehy drogenase (LDH), effusion LDH/effusion ADA, and serum white blood cell (WBC) were identified as valuable parameters used for developing a nomogram. The nomogram showed a good diagnostic performance in the training set. A novel scoring system was then established based on the nomogram to distinguish TPE from non-TB BPE. The scoring system showed good diagnostic performance in the training set [area under the curve (AUC) (95% confidence interval (CI)), 0.937 (0.917–0.957); sensitivity, 89.0%, and specificity, 89.5%], the internal validation set [AUC (95%CI), 0.934 (0.902–0.966); sensitivity, 88.7%, and specificity, 90.3%], and the external validation set [(AUC (95%CI), 0.941 (0.891–0.991); sensitivity, 93.6%, and specificity, 87.5%)], respectively.

**Conclusions:**

The study developed and validated a novel scoring system based on a nomogram originated from six clinical parameters. The novel scoring system showed a good diagnostic performance in distinguishing TPE from non-TB BPE and can be conveniently used in clinical settings.

**Supplementary Information:**

The online version contains supplementary material available at 10.1186/s12931-022-02051-4.

## Background

Tuberculosis (TB) remains the most common cause of death from a single infectious pathogen worldwide in 2019 [1]. It is estimated that with 10 million people developed TB disease and 1.4 million TB patients died in 2019 [1]. Tuberculous pleural effusion (TPE) is a common clinical manifestation of extra-pulmonary TB, which accounts for 25 ~ 30% of total TB cases in TB-endemic regions, including China [2–4]. Early and accurate diagnosis of TPE is extremely critical for the management of the disease. Currently, the gold standards for TPE diagnosis were based on the detection of acid-fast bacilli (AFB) in sputum, pleural fluid, or pleural biopsy tissues through *Mycobacterium tuberculosis* (*M. tuberculosis*) culture or performed by thoracoscopy [4, 5]. However, the limited sensitivity, low accuracy and invasive examination of those diagnostic tools compromised their diagnostic value in clinical practice [6–8]. Alternative diagnostic methods, including tuberculin skin test (TST), adenosine deaminase (ADA), and interferon-gamma release assays (IGRAs), have improved the speed for TPE diagnosis in recently years [4, 9–11]. However, the sensitivity and/or specificity of those methods were still insufficient when separated TPE from other type of pleural effusion (PE), such as malignant pleural effusion (MPE) and parapneumonic pleural effusion (PPE) [9–11].

Therefore, it was urgent to seek and establish a highly sensitive, accurate and less invasive diagnostic marker or method for TPE patients. The aim of this study was to construct a scoring system based on a nomogram to distinguish TPE from non-TB BPE. Besides, we also validated the diagnostic performance of the developed scoring system in the internal set and the external set from our patients and another hospital, retrospectively.

## Materials and methods

### Patients and study design

**Fig. 1:**
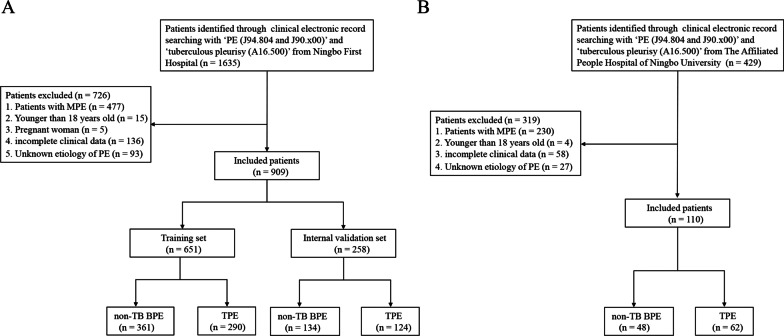
The flowchart of patient selection. **A** Ningbo First Hospital set. **B** The Affiliated People Hospital of Ningbo University set. *MPE* malignant pleural effusion, *PE* pleural effusion, *BPE* benign pleural effusion, *TB* tuberculosis

This was a retrospective study of individuals more than 18 years old who were admitted to Ningbo First Hospital with newly diagnosed PE between January 2014 and March 2021. A flow diagram of patient selection was presented in Fig. 1. We retrospectively reviewed all consecutive patients with the keyword ‘PE (J94.804 and J90. × 00)’ and ‘tuberculous pleurisy (A16.500)’ in the clinical electronic record system of Ningbo First Hospital. All the patients were first admitted to our hospital because of pleural effusion. All PE samples and concomitant blood samples were taken and tested for counts and biochemical parameters. The data from the first sample of PE and blood obtained in each patient was considered for analysis. The related demographic, laboratory, and clinical information for each patient were extracted from the clinical electronic record system. Finally, a total of 909 patients with BPE were enrolled in this study. Patients were randomly separated as the training set (n = 651) and the internal validation set (n = 258) at a 7:3 ratio, A cohort of 110 patients with PE in the Affiliated People Hospital of Ningbo University from August 2020 to November 2021 were used as the external validation set. Among 909 patients, 414 patients with BPE were caused by tuberculous pleurisy (TBP), and 495 patients were caused by parapneumonic effusion (PPE), chronic heart failure (CHF), empyema, parasitic infection and so on. Patients that meet all the following criteria were included: (i) PE was diagnosed underwent either ultrasonography, chest CT, or X-ray (ii) patients underwent diagnosis for PE by cytology, thoracentesis or pleural biopsy and follow-up (at least 6 months). The exclusion criteria were as follows: (i) patients diagnosed with MPE; (ii) age < 18 years old; (iii) pregnant women; (iv) patients with incomplete clinical data; (v) unknown etiology of PE.

The primary aim of the present study was to develop a scoring system with high predictive accuracy to accurately differentiate TPE from non-TPE. The training set included 70% of the patients with PE from Ningbo First Hospital to develop a novel scoring system based on a nomogram to distinguish patients with TPE from patients with non-TPE. The internal validation set included the remaining 30% patients with PE from Ningbo First Hospital to validate the diagnostic performance of the scoring system. The external validation set included 110 patients with PE from Affiliated People Hospital of Ningbo University, independent of the patients from Ningbo First Hospital, were used to further validate the predictive model.

This study was approved by the Ethics Committee of Ningbo First Hospital and the Affiliated People Hospital of Ningbo University. This study was conducted in accordance with the Helsinki Declaration. The requirement for written informed consent was exempted because of the retrospective nature.

### Diagnostic criteria for BPE and TPE

BPE was diagnosed based on the following criteria: (a) no tumor cells found in PE; (b) PE of a known etiology, such as TPE or parapneumonic PE, that vanished after optimal treatment; (c) no signs of malignant disease were developed during the follow-up. TPE patients who were first diagnosed and treated in our hospital were included in our study, and was diagnosed based on any of the following criteria: (a) *M. tuberculosis* was positive in culture of the pleural effusion or pleura tissue; (b) granulomatous inflammation was present in the pleura biopsy by histologic examination and *M. tuberculosis* was isolated from other sites; or (c) the both presence of granulomatous inflammation in the pleura biopsy by histologic examination and clinical response to anti-TB treatment [12–14].

### Data collection

The following clinical and laboratory data were acquired from the clinical electronic record system, including age, gender, smoking history, effusion routine [effusion white blood cell (WBC), neutrophil count, and lymphocyte count], effusion biochemical indexes [total protein, glucose, ADA, and lactatedehy drogenase (LDH)], blood routine (WBC, neutrophil count, and lymphocyte count), blood indexes [high-sensitivity C-reactive protein (hsCRP), erythrocyte sedimentation rate (ESR), ADA, and LDH], carbohydrate antigen 125 (CA125), and carbohydrate antigen 19–9 (CA19-9) in PE and serum.

### Statistical analysis

Continuous variables were presented as median and inter quartile rang (IQR, 25th–75th), and were compared using either a t-test or Mann–Whitney *U* test, as appropriate. Categorical variables were presented as number and percentage (n, %), and were compared using the Chi-square (X^2^) test or Fisher’s exact test. Univariate logistic regression analysis was used to screen the independent factors in the training set, and all variables at a significant level [area under the curve (AUC) > 0.6] were selected for multivariate logistic analysis. Then, stepwise selection using the Akaike information criterion (AIC) in the multivariable logistic regression models determined the statistically significant variables. Odds ratios (ORs) were estimated and presented with 95% confidence intervals (CI). Selected variables were incorporated into the nomograms to construct the scoring system using the rms package of R. Calibration curves and decision curve analysis (DCA) were also performed. Receiver operating characteristic (ROC) curve and the corresponding AUCs were calculated to determine the discrimination capacity of the models in distinguishing TPE from non-TB BPE. Besides, the sensitivity, specificity, positive predictive value (PPV), negative predictive value (NPV), positive likelihood ratio (PLR), and negative likelihood ratio (NLR) were performed to assess the diagnostic accuracy of the nomogram in the training set and validation sets. All statistical analyses were performed using R (packages rms, MASS, OptimalCutpoints, pROC, and rmda; version 4.0.5; http://www.r-project.org) and SPSS 22.0 (SPSS Inc., Chicago, IL USA). Two-sided *P* < 0.05 was considered to be significant.

## Results

### Baseline characteristics

A total of 909 patients with PE from Ningbo First Hospital were included in the present study, and were randomly divided into the training set (n = 651) and the internal validation set (n = 258), respectively. Besides, 110 patients from the Affiliated People Hospital of Ningbo University were included in the external validation set. The demographic and clinical, and laboratory characteristics of the patients among the three groups were presented in Table 1.

**Table 1:** The clinical characteristics of the training set, internal validation set, and external validation set

Characteristics	Training set (n = 651)		Internal validation set (n = 258)		External validation set (n = 110)	
	Non-TB BPE (n = 361)	TPE (n = 290)	Non-TB BPE (n = 134)	TPE (n = 124)	Non-TB BPE (n = 48)	TPE (n = 62)
Age (years)	67.0 (55.5–79.0)	48.0 (28.0–67.3)	66.0 (55.0–76.3)	42.0 (28.0–58.8)	66.5 (57.8–79.0)	51.0 (34.0–69.0)
Gender (n, %)						
Female	116 (32.1)	87 (30.0)	47 (35.1)	44 (35.5)	8 (16.7)	17 (27.4)
Male	245 (67.9)	203 (70.0)	87 (64.9)	80 (64.5)	40 (83.3)	45 (73.6)
Smoke status (n, %)						
Non-smokers	216 (59.8)	186 (64.1)	86 (64.2)	87 (70.2)	27 (56.2)	41 (66.1)
C/F smokers	145 (40.2)	104 (35.9)	48 (35.8)	37 (29.8)	21 (43.8)	21 (33.9)
Effusion						
WBC (× 10.^9^/L)	1.27 (0.42–3.85)	2.18 (1.22–3.70)	1.50 (0.40–5.17)	2.39 (1.42–3.89)	0.88 (0.4–2.93)	1.36 (0.57–2.95)
Neutrophil (× 10.^9^/L)	0.23 (0.06–1.50)	0.14 (0.05–0.38)	0.25 (0.04–1.35)	0.18 (0.06–0.44)	0.24 (0.06–1.23)	0.13 (0.04–0.25)
Lymphocyte (× 10.^9^/L) Total protein (g/L)	0.39 (0.14–1.21) 39.50 (25.25–48.80)	1.86 (0.95–2.95) 51.25 (48.00–54.30)	0.49 (0.15–1.57) 40.25 (26.30–51.93)	1.99 (1.12–3.28) 51.75 (48.25–55.23)	0.33 (0.12–1.21) 40.85 (25.55–48.40)	1.10 (0.36–2.30) 49.10 (45.18–52.53)
Glucose (mmol/L)	6.82 (4.89–8.54)	5.39 (4.30–6.38)	6.50 (4.77–8.48)	5.55 (4.36–6.37)	7.06 (5.04–9.41)	5.52 (4.48–7.00)
ADA (U/L)	10.50 (5.20–20.85)	42.25 (32.50–54.83)	10.00 (4.40–21.42)	44.00 (33.38–55.83)	12.00 (5.00–23.00)	43.50 (32.75–53.00)
LDH (U/L)	228.0 (121.0–755.5)	441.0 (309.5–666.8)	301.0 (126.5–889.5)	450 (318.8–642.8)	387.5 (189.8–997.3)	506.5 (361.0–842.3)
CA125 (U/ml)	1134.5 (546.9–2029.2)	995.1 (452.2–1638.7)	1096.5 (490.0–1855.0)	1031.0 (464.8–1630.4)	1205.7 (492–2102.5)	897.8 (437.9–1757.8)
CA19-9 (U/ml)	2.00 (1.00–3.63)	2.50 (1.29–4.10)	2.00 (0.98–4.95)	2.50 (1.51–4.58)	2.15 (1.03–3.85)	2.20 (1.08–4.75)
Serum						
WBC (× 10.^9^/L)	7.50 (5.60–10.50)	6.18 (5.06–7.67)	7.52 (5.86–10.48)	6.40 (5.10–7.63)	6.55 (5.35–8.90)	5.95 (4.30–7.30)
Neutrophil (× 10.^9^/L)	5.10 (3.10–8.10)	4.20 (3.30–5.50)	5.15 (4.00–7.65)	4.28 (3.40–5.44)	4.74 (3.78–6.98)	4.36 (2.80–5.51)
Lymphocyte (× 10.^9^/L)	1.10 (0.70–1.50)	1.10 (0.80–1.40)	1.10 (0.80–1.50)	1.10 (0.80–1.42)	0.99 (0.69–1.38)	0.91 (0.56–1.24)
hsCRP (mg/L)	27.04 (6.28–105.27)	44.48 (17.22–79.25)	30.54 (5.87–107.69)	42.84 (19.07–68.87)	56.68 (13.49–163.5)	68.80 (28.23–93.80)
ESR (mm/h)	47.0 (22.5–69.0)	46.0 (27.0–69.0)	43.5 (19.0–66.3)	46.5 (33.0–65.0)	54.00 (25.00–73.50)	46.50 (30.00–65.25)
ADA (U/L)	10.60 (7.80–14.80)	11.55 (8.68–14.80)	10.60 (7.70–13.53)	11.70 (9.20–15.25)	9.00 (7.00–13.00)	11.50 (9.00–15.00)
LDH (U/L)	187.0 (156.0–232.5)	193.0 (165.0–239.0)	199.0 (160.8–245.3)	185.0 (156.0–226.0)	202.0 (163.0–250.8)	176.5 (161.8–207.0)
CA125 (U/ml)	63.8 (28.4–148.7)	103.3 (55.7–183.8)	61.3 (30.1–129.7)	124.1 (54.1–192.1)	72.3 (42.2–169.6)	111.7 (64.4–211.4)
CA19-9 (U/ml)	8.90 (4.25–16.20)	6.09 (3.18–10.53)	6.50 (3.98–15.50)	5.80 (3.60–10.30)	8.80 (4.30–13.00)	4.20 (2.18–9.40)

*TB* tuberculous, *WBC* white blood cell, *ADA* adenosine deaminase, *LDH* lactatedehy drogenase, *CA125* carbohydrate antigen 125, *CA19-9* carbohydrate antigen 19-9, *hsCRP* high-sensitivity C-reactive protein, *ESR* erythrocyte sedimentation rate

Continuous variables were presented as median and inter quartile rang (IQR, 25th–75th). Categorical variables were presented as number and percentage (n, %)

### Univariate and multivariate logistic regression analyses in patients with TPE and non-TB BPE

Additional file 1: Table S1 compared the demographic, clinical, and laboratory variables between TPE and non-TB BPE in the training set. The cutoff values of those variables were calculated using the Youden index. As shown in Additional file 2: Table S2, most of the included variables were significantly different between the patients with TPE and non-TB BPE. The results calculated by univariate logistic analysis were shown in Additional file 2: Table S2. All 24 variables showed statistical significance. To establish an accurate prediction model, 16 variables with an AUC > 0.6 were performed to multivariate regression analysis. Stepwise selection using AIC method in the regression model identified six most valuable variables in distinguishing TPE from non-TB BPE with highest order. Table 2 summarized the results of the multivariate logistic regression analysis. Results were as follows: age (OR (95%CI), 0.419 (0.232–0.755)), effusion lymphocyte (OR (95%CI), 3.229 (1.824–5.715)), effusion ADA (OR (95%CI), 7.258 (3.745–14.066)), effusion LDH (OR (95%CI), 6.626 (2.894–15.172)), effusion LDH/ADA (OR (95%CI), 0.189 (0.097–0.370)), and serum WBC (OR (95%CI), 0.331 (0.173–0.634)) (Table 2).

**Table 2:** Multivariate logistic regression analysis of the clinical characteristics in the training set

Variables	Multivariate analysis OR (95%CI) *P* value	
Age (years)		
< 54	0.419 (0.232–0.755)	0.004
≥ 54		
Effusion lymphocyte (× 10.^9^/L)		
< 0.80	3.229 (1.824–5.715)	< 0.001
≥ 0.80		
Effusion ADA (U/L)		
< 22.75	7.258 (3.745–14.066)	< 0.001
≥ 22.75		
Effusion LDH (U/L)		
< 247.5	6.626 (2.894–15.172)	< 0.001
≥ 247.5		
Effusion LDH/ADA		
< 17.07	0.189 (0.097–0.370)	< 0.001
≥ 17.07		
Serum WBC (× 10.^9^/L)		
< 8.68	0.331 (0.173–0.634)	0.001
≥ 8.68		

*OR* odds ratio, *CI* confidence interval, *ADA* adenosine deaminase, *LDH* lactatedehy drogenase, *WBC* white blood cell, effusion *LDH/ADA* effusion LDH/ effusion ADA

### Development and validation of the nomogram prediction model

**Fig. 2:**
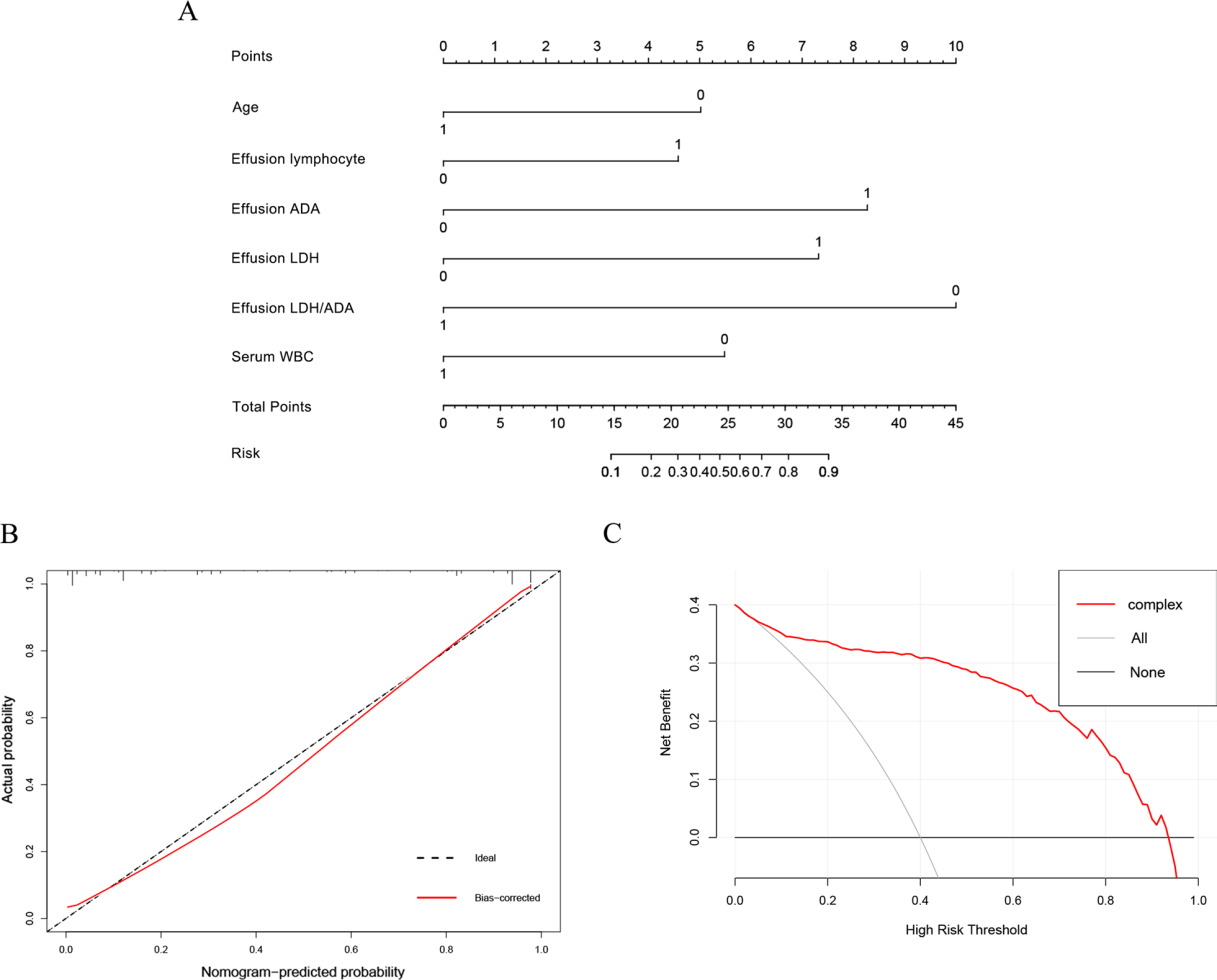
Development of the diagnostic nomogram. **A** Diagnostic nomogram for distinguishing TPE from non-TB BPE in the training set. **B** Calibration curve of the nomogram. **C** Decision curve analysis of the nomogram

A nomogram based on the above six variables was developed and presented in Fig. 2A. The calibration curve of the nomogram showed that the predicted line overlapped well with the reference line, indicating a good performance of the diagnostic monogram in the training set (Fig. 2B). In addition, the DCA was applied to assess the net benefit of the diagnostic nomogram in order to verify the clinically utility of the model. Results showed that patients would benefit more over the “treat-all” or “treat-none” strategy when the threshold probability was > 0.4 (Fig. 2C).

### Diagnostic performance of the scoring system in the training set and validation sets

**Fig. 3:**
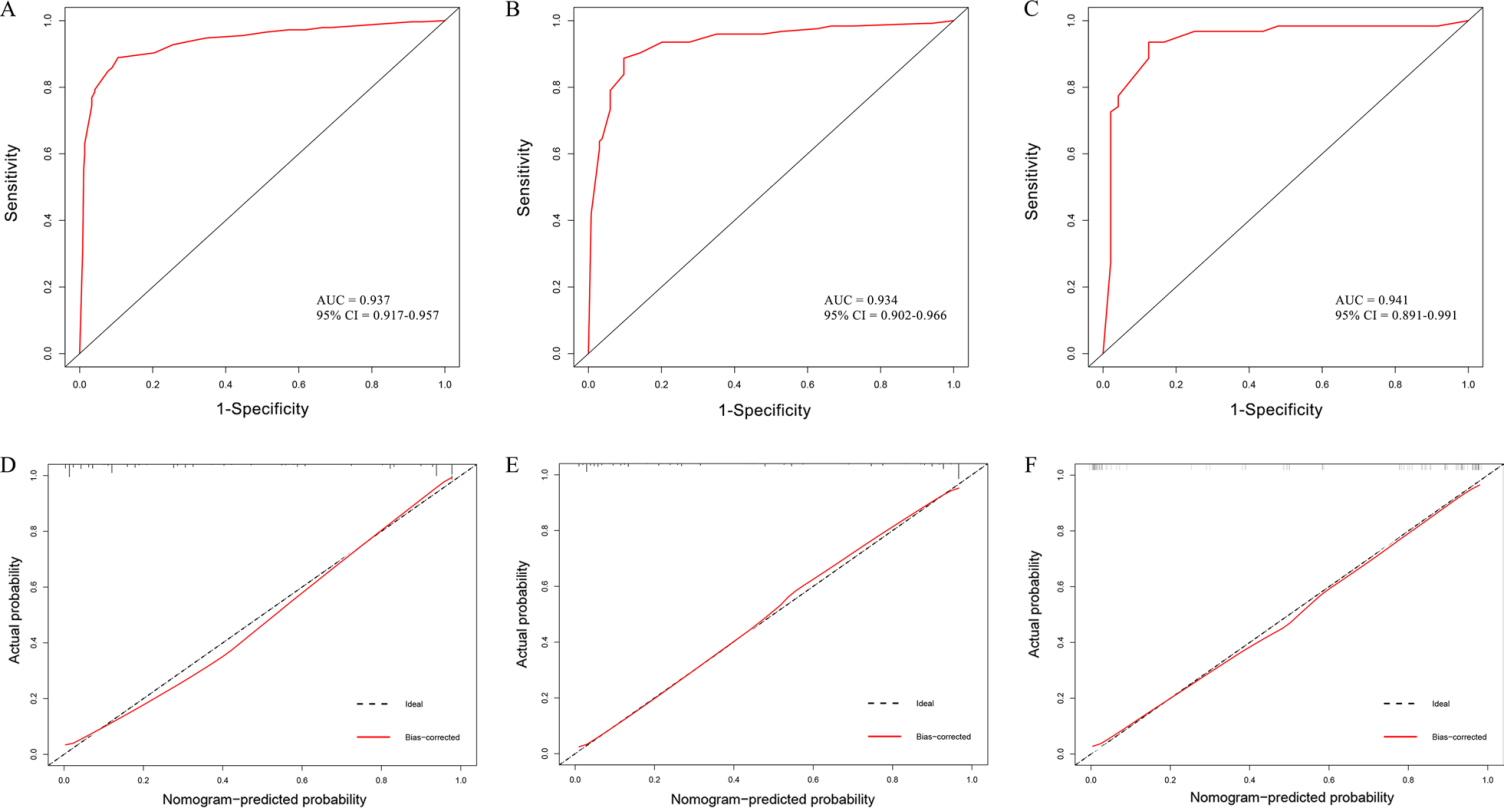
Discrimination and calibration of the scoring system for distinguishing TPE from non-TB BPE. **A**–**C** ROC curves of the scoring system in the training set, internal validation set, and external validation set. **B**–**D** Calibration curves of the scoring system in the training set, internal validation set, and external validation set

In the training set, effusion LDH/ADA showed the largest impact on the discrimination of TPE from non-TB BPE in the model with a point of 10 (Fig. 2A). The other five variables were then modified to integer points: age (5 points), effusion lymphocyte (5 points), effusion ADA (8 points), effusion LDH (7 points), effusion and serum WBC (6 points) (Table 3). The optimal cutoff value for the total scores was calculated using ROC. When the cutoff value was 23 points, this scoring system showed a good discriminative performance in distinguishing TPE from non-TB BPE with an AUC of 0.937 (95%CI, 0.917–0.957, Fig. 3A and Table 4). The corresponding specificity, sensitivity, PLR, NLR, PPV, and NPV values were 89.0%, 89.5%, 8.5, 0.12, 87.2%, and 91.2%, respectively (Table 4).

**Table 3:** Diagnostic nomogram score calculation for the training set

Parameters	Score generated from nomogram (points)	Score modified from nomogram (points)
Age (< 54 years)	5	5
Effusion lymphocyte (≥ 0.80 × 10.^9^/L)	4.58	5
Effusion ADA (≥ 22.75 U/L)	8.25	8
Effusion LDH (≥ 247.5 U/L)	7.3	7
Effusion LDH/ADA (< 17.07)	10	10
Serum WBC (< 8.68 × 10.^9^/L)	5.5	6

*ADA* adenosine deaminase, *LDH* lactatedehy drogenase, *WBC* white blood cell, *LDH/ADA* effusion LDH/ effusion ADA

**Table 4:** Diagnostic performance of the scoring system based on nomogram in differentiating TPE from non-TB BPE in the training set and validation sets

Variables	Training set	Internal validation set	External validation set
AUC (95%CI)	0.937 (0.917–0.957)	0.934 (0.902–0.966)	0.941 (0.891–0.991)
Sensitivity (95%CI)	89.0% (84.8–92.3%)	88.7% (81.8–93.7%)	93.6% (84.3–98.2%)
Specificity (95%CI)	89.5% (85.8–92.4%)	90.3% (84.0–94.7%)	87.5% (74.8–95.3%)
PLR (95%CI)	8.5 (6.2–11.4)	9.1 (5.4–15.4)	7.5 (3.5–15.9)
NLR (95%CI)	0.12 (0.09–0.21)	0.13 (0.08–0.18)	0.07 (0.03–0.20)
PPV (95%CI)	87.2% (83.4–90.2%)	89.4% (83.4–93.4%)	90.6% (82.0–95.3%)
NPV (95%CI)	91.0% (87.9–93.3%)	89.6% (84.0–93.4%)	91.3% (80.2–96.5%)

*TPE* tuberculous pleural effusion, *BPE* benign pleural effusion, *AUC* area under curve, *CI* confidence interval, *PLR* positive likelihood ratio, *NLR* negative likelihood ratio, *PPV* positive predictive value, *NPV* negative predictive value

The scoring system also exhibited good discriminative values in distinguishing TPE from non-TB BPE in the internal validation set and external validation set, with AUCs of 0.934 (95%CI, 0.902–0.966, Fig. 3B and Table 4) and 0.941 (95%CI, 0.891–0.991, Fig. 3C and Table 4), respectively. The specificity, sensitivity, PLR, NLR, PPV, and NPV values in the internal validation set were 88.7%, 90.3%, 9.1, 0.13, 89.4%, and 89.6%, respectively (Table 4). The specificity, sensitivity, PLR, NLR, PPV, and NPV values in the external validation set were 93.6%, 87.5%, 7.5, 0.07, 90.6%, and 91.3%, respectively (Table 4). Furthermore, the calibration curve of the scoring system also showed good agreements in the three datasets (Fig. 3D–F).

## Discussion

Early diagnosis and prompt therapy for patients with TPE is critical to prevent severe complications (pleural thickening, empyema, and calcification, etc*.*) and mortality. Despite the availability of various diagnostic methods, the early differential diagnosis of TPE from MPE and other non-TB BPE remains to be challenging in clinical practice. Besides, paucibacillary nature of the disease, inappropriate and inadequate test samples, ineffective conventional microbiological techniques, lack of thoracoscopy equipment all lead to the difficulty for diagnosing TPE.

Conventional histopathologic presence of *M. tuberculosis* on culture, or pleural pathology showing caseating granuloma is the gold standard for diagnosing TPE, however, the diagnostic tests were time consuming and low positive rate [8, 11]. Tuberculin skin test (TST) and interferon-gamma release assays (IGRAs) were two common detection methods for diagnosing TPE, but the limitations of inaccuracy, inconsistent sensitivity, and time to diagnosis have retained its efficacies [11, 15, 16]. Under the circumstances, thoracoscopy seemed to provide a higher sensitivity (93–100%) and accuracy for diagnosing TPE, however, it was an invasive and expensive diagnostic method with a reported 2–6% rate of complications [8, 17, 18]. The common complications were bleeding, fever, empyema, pneumonia, and prolonged air leak and so on [18]. Besides, several patients with underlying disease progression and elderly patients cannot tolerate the examination.

In recently years, the Xpert MTB/RIF (Xpert) and/or next-generation Xpert MTB/RIF Ultra (Xpert Ultra), two nucleic acid detection methods, have been increasingly used to diagnose pulmonary TB, rifampicin (RIF) resistance as well as extra-pulmonary TB in various types of clinical specimens endorsed by World Health Organization (WHO) [19, 20]. A meta-analysis indicated that the pooled sensitivity of Xpert in diagnosing TPE was only 51.4% [21]. The low sensitivity has compromised its diagnostic capacity for TPE, which might be attributed to the number of mycobacteria and performance of amplification techniques. Therefore, an effective and noninvasive diagnostic method is urgently needed for diagnosing and management of TPE.

Nomograms are a graphical representation of a complex mathematical formula, which are widely used to estimate diagnosis and prognosis for a variety of diseases by integrating clinical, biologic, and/or genetic variables in medicine [22]. Previously, we and other investigators had reported the application of nomogram in differentiating MPE from BPE [23, 24]. In the present study, we developed a scoring system based on a nomogram to distinguish TPE from non-TB BPE. We initially integrated 26 variables, including not only primary clinical and laboratory variables but calculated ratios. We selected six most significant variables (age, effusion lymphocyte, effusion ADA, effusion LDH, effusion LDH/ADA, and serum WBC) analyzed by multivariate regression analysis to construct a predictive model. Our model showed a good diagnostic performance in distinguishing TPE from non-TB BPE in the derivation and validation sets. The integrated six commonly indexes were inexpensive, routinely tested, and readily available in most hospitals, therefore, our model is convenient to apply in clinical practice.

Effusion ADA has long been used to diagnose TPE in numerous studies [11, 15]. Michot et al. indicated that effusion ADA at an optimal value of 41.5 U/L might be a useful biomarker to differentiate TPE from non-TPE with a sensitivity and specificity were with a sensitivity of 97.1% and a specificity of 92.9% [25]. A study conducted by Garcia-Zamalloa et al. showed a similar cutoff value of effusion ADA with 40U/L [26]. However, a recent study from China showed that best cutoff value of effusion ADA for TBP was 27U/L with a sensitivity of 81% and a specificity of 78% [27]. A similar cutoff value of effusion ADA was also found in our study (22.75 U/L). Therefore, the optimal cutoff values are still controversial due to the prevalence rates of the disease, sample sizes, different test methods, or HIV co-infection [11]. Besides, a similar or even higher level of effusion ADA has been reported in PPE, especially in patients with empyema [28, 29]. Effusion LDH was recommended to assist in the classification of patients with complicated parapneumonic effusion (CPPE) [30]. However, an elevated effusion LDH in TPE, PPE, and MPE and the low sensitivity and specificity of LDH in differentiating TPE from PPE limited its utility in clinical practice [30].

The effusion LDH/ADA ratio was also assessed in differentiating TPE from PPE. Wang et al. indicated that effusion LDH/ADA ratio might be a useful biomarker in diagnosing TPE at a cut-off level of 16.20, with a sensitivity of 93.62% and a specificity of 93.06% [31]. Another study from New Zealand also showed that effusion LDH/ADA ratio at a cutoff value of 15 demonstrated a high sensitivity and specificity in distinguishing TPE from non-TB effusion [32]. Similarly, our study showed a cutoff value of 17.07 for effusion LDH/ADA. Further prospective investigations were needed to validate the results in the future.

To our knowledge, this was the first study to evaluate a scoring system based on a nomogram in distinguishing TPE from non-TB BPE. The developed scoring system might be reliable and accuracy in distinguishing TPE from non-TB BPE, which was assessed by the indexes of sensitivity, specificity, PLR, NLR, PPV, and NPV in the training and validation sets. Our study incorporated the most common and valuable indexes in the predictive model to differentiating TPE from non-TB BPE, which was better than any single variable alone. The six easily accessible and inexpensive variables routinely tested and acquired in most hospitals. Therefore, our diagnostic model for differentiating TPE from non-TB BPE could be easily used in clinical practice in most hospitals, especially in primary hospitals.

Our study had some limitations. First, the present study was retrospective design. Only routine biomarkers in serum and PE were included in the study. Several newly potential biomarkers, such as interleukin 27 (IL-27) and tumor necrosis factor-α (TNF-α), might provide better diagnostic accuracy. Second, external validation was a single-center with a small sample size. Third, our nomogram did not incorporate imaging data into the scoring system, which might be useful. Besides, we also did not compare the diagnostic accuracy of our scoring system and other diagnostic tests for unavailable data, such as IGRAs and Xpert Ultra. Finally, this study was conducted on Chinese patients. Since the incidence of TB differs from country to country, the results of this study cannot be applied to patients in other countries. Further multicentric and prospective investigations containing comprehensive data was needed to validate our results.

## Conclusions

Taken together, the present study developed a novel scoring system based on a nomogram with six clinical and laboratory variables to aid differential diagnosis of TPE and non-TB TPE. Our novel scoring system showed a good diagnostic performance and calibration in distinguishing TPE from non-TB TPE in the training set and the validation sets. Further multicentric and prospective investigations should be used to validate the accessible and non-invasive nomogram.

## Supplementary Information

**Additional file 1: Table S1.** The clinical characteristics between non-TB BPE and TPE in the training set.

**Additional file 2: Table S2.** Univariate logistic regression analysis of the clinical characteristics in the training set.

## Data Availability

The data that support the findings of this study are available from the corresponding author upon reasonable request.

## Abbreviations

TPE: Tuberculous pleural effusion
TB: Tuberculosis
BPE: Benign pleural effusion
ADA: Adenosine deaminase
LDH: Lactatedehy drogenas
WBC: White blood cell
AUC: Area under the curve
AFB: Acid-fast bacilli
*M. tuberculosis*: *Mycobacterium tuberculosis*
TST: Tuberculin skin test
IGRAs: Interferon-gamma release assays
PE: Pleural effusion
MPE: Malignant pleural effusion
PPE: Parapneumonic pleural effusion
TBP: Tuberculous pleurisy
CHF: Chronic heart failure
BALF: Bronchoalveolar lavage fluid
hsCRP: High-sensitivity C-reactive protein
ESR: Erythrocyte sedimentation rate
CA125: Carbohydrate antigen 125
CA19-9: Carbohydrate antigen 19-9
SD: Standard deviation
AIC: Akaike information criterion
ORs: Odds ratios
CI: Confidence intervals
DCA: Decision curve analysis
ROC: Receiver operating characteristic
PPV: Positive predictive value
NPV: Negative predictive value
PLR: Positive likelihood ratio
NLR: Negative likelihood ratio
IL-27: Interleukin 27
TNF-α: Tumor necrosis factor-α

## References

[1] Chakaya J, Khan M, Ntoumi F, Aklillu E, Fatima R, Mwaba P (2021). Global Tuberculosis Report 2020—reflections on the global TB burden, treatment and prevention efforts. Int J Infect Dis.

[2] Diacon AH, Van de Wal BW, Wyser C, Smedema JP, Bezuidenhout J, Bolliger CT (2003). Diagnostic tools in tuberculous pleurisy: a direct comparative study. Eur Respir J.

[3] Light RW (2010). Update on tuberculous pleural effusion. Respirology.

[4] Zhai K, Lu Y, Shi HZ (2016). Tuberculous pleural effusion. J Thorac Dis.

[5] Vorster MJ, Allwood BW, Diacon AH (2015). Koegelenberg CF Tuberculous pleural effusions: advances and controversies. J Thorac Dis.

[6] Agarwal R, Aggarwal AN, Gupta D (2013). Diagnostic accuracy and safety of semirigid thoracoscopy in exudative pleural effusions: a meta-analysis. Chest.

[7] Wang XJ, Yang Y, Wang Z, Xu LL, Wu YB, Zhang J (2015). Efficacy and safety of diagnostic thoracoscopy in undiagnosed pleural effusions. Respiration.

[8] Shaikh F, Lentz RJ, Feller-Kopman D, Maldonado F (2020). Medical thoracoscopy in the diagnosis of pleural disease: a guide for the clinician. Expert Rev Respir Med.

[9] Liu Y, Ou Q, Zheng J, Shen L, Zhang B, Weng X (2016). A combination of the QuantiFERON-TB gold in-tube assay and the detection of adenosine deaminase improves the diagnosis of tuberculous pleural effusion. Emerg Microbes Infect.

[10] Chung JH, Han CH, Kim CJ, Lee SM (2011). Clinical utility of QuantiFERON-TB GOLD In-Tube and tuberculin skin test in patients with tuberculous pleural effusions. Diagn Microbiol Infect Dis.

[11] Gopi A, Madhavan SM, Sharma SK, Sahn SA (2007). Diagnosis and treatment of tuberculous pleural effusion in 2006. Chest.

[12] Lin MT, Wang JY, Yu CJ, Lee LN, Yang PC (2009). *Mycobacterium tuberculosis* and polymorphonuclear pleural effusion: incidence and clinical pointers. Respir Med.

[13] Bielsa S, Palma R, Pardina M, Esquerda A, Light RW, Porcel JM (2013). Comparison of polymorphonuclear- and lymphocyte-rich tuberculous pleural effusions. Int J Tuberc Lung Dis.

[14] Villena Garrido V, Cases Viedma E, Fernández Villar A, de Pablo GA, Pérez Rodríguez E, Porcel Pérez JM (2014). Recommendations of diagnosis and treatment of pleural effusion. Update. Arch Bronconeumol.

[15] Keng LT, Shu CC, Chen JY, Liang SK, Lin CK, Chang LY (2013). Evaluating pleural ADA, ADA2, IFN-γ and IGRA for diagnosing tuberculous pleurisy. J Infect.

[16] Jiang J, Shi HZ, Liang QL, Qin SM, Qin XJ (2007). Diagnostic value of interferon-gamma in tuberculous pleurisy: a metaanalysis. Chest.

[17] Wang Z, Xu LL, Wu YB, Wang XJ, Yang Y, Zhang J (2015). Diagnostic value and safety of medical thoracoscopy in tuberculous pleural effusion. Respir Med.

[18] Rahman NM, Ali NJ, Brown G, Chapman SJ, Davies RJ, Downer NJ (2010). Local anaesthetic thoracoscopy: British Thoracic Society Pleural Disease Guideline 2010. Thorax.

[19] WHO Guidelines Approved by the Guidelines Review Committee. In Automated real-time nucleic acid amplification technology for rapid and simultaneous detection of tuberculosis and rifampicin resistance: Xpert MTB/RIF assay for the diagnosis of pulmonary and extrapulmonary TB in Adults and Children: Policy Update. Geneva: World Health Organization. Copyright © World Health Organization 2013; 2013. 25473701

[20] Chakravorty S, Simmons AM, Rowneki M, Parmar H, Cao Y, Ryan J, et al. The New Xpert MTB/RIF Ultra: improving detection of *Mycobacterium tuberculosis* and resistance to rifampin in an assay suitable for point-of-care testing. mBio. 2017; 8. 10.1128/mBio.00812-17 PMC5574709 28851844

[21] Sehgal IS, Dhooria S, Aggarwal AN, Behera D, Agarwal R (2016). Diagnostic performance of Xpert MTB/RIF in tuberculous pleural effusion: systematic review and meta-analysis. J Clin Microbiol.

[22] Balachandran VP, Gonen M, Smith JJ, DeMatteo RP (2015). Nomograms in oncology: more than meets the eye. Lancet Oncol.

[23] Wang S, Tian S, Li Y, Zhan N, Guo Y, Liu Y (2020). Development and validation of a novel scoring system developed from a nomogram to identify malignant pleural effusion. EBioMedicine.

[24] Wu A, Liang Z, Yuan S, Wang S, Peng W, Mo Y (2021). Development and validation of a scoring system for early diagnosis of malignant pleural effusion based on a nomogram. Front Oncol.

[25] Michot JM, Madec Y, Bulifon S, Thorette-Tcherniak C, Fortineau N, Noël N (2016). Adenosine deaminase is a useful biomarker to diagnose pleural tuberculosis in low to medium prevalence settings. Diagn Microbiol Infect Dis.

[26] Garcia-Zamalloa A, Taboada-Gomez J (2012). Diagnostic accuracy of adenosine deaminase and lymphocyte proportion in pleural fluid for tuberculous pleurisy in different prevalence scenarios. PLoS ONE.

[27] Lei X, Wang J, Yang Z, Zhou S, Xu Z (2019). Diagnostic value of pleural effusion mononuclear cells count and adenosine deaminase for tuberculous pleurisy patients in China: a case-control study. Front Med (Lausanne).

[28] Manuel Porcel J, Vives M, Esquerda A, Ruiz A (2006). Usefulness of the British Thoracic Society and the American College of Chest Physicians guidelines in predicting pleural drainage of non-purulent parapneumonic effusions. Respir Med.

[29] Porcel JM, Esquerda A, Bielsa S (2010). Diagnostic performance of adenosine deaminase activity in pleural fluid: a single-center experience with over 2100 consecutive patients. Eur J Intern Med.

[30] Davies CW, Gleeson FV, Davies RJ (2003). BTS guidelines for the management of pleural infection. Thorax.

[31] Wang J, Liu J, Xie X, Shen P, He J, Zeng Y (2017). The pleural fluid lactate dehydrogenase/adenosine deaminase ratio differentiates between tuberculous and parapneumonic pleural effusions. BMC Pulm Med.

[32] Blakiston M, Chiu W, Wong C, Morpeth S, Taylor S. Diagnostic performance of pleural fluid adenosine deaminase for tuberculous pleural effusion in a low-incidence setting. J Clin Microbiol. 2018; 56. 10.1128/JCM.00258-18 PMC6062806 29793967

