# Supplementary material for: Development and validation of a prediction model for tuberculous pleural effusion: a large cohort study and external validation
Source: Respir Res. 2022 May 27;23:134. doi: 10.1186/s12931-022-02051-4 (PMC9145463; doi:10.1186/s12931-022-02051-4)
Supplement: Supplementary file 1 — Additional file 1: Table S1. The clinical characteristics between non-TB BPE and TPE in the training set. [file 12931_2022_2051_MOESM1_ESM.docx]

Table S1 The clinical characteristics between non-TB BPE and TPE in the training set

| Variables |  | N | non-TB BPE (n, %) | TPE (n, %) | *P* value |
| --- | --- | --- | --- | --- | --- |
| Age (years) | < 54 | 255 | 82 (22.7) | 173 (59.7) | < 0.001 |
|  | ≥ 54 | 396 | 279 (77.3) | 117 (40.3) |  |
| Gender | Female | 203 | 116 (32.1) | 87 (30.0) | 0.610 |
|  | Male | 448 | 245 (67.9) | 203 (70.0) |  |
| Smoking status | Non-smokers | 402 | 216 (59.8) | 186 (64.1) | 0.379 |
|  | C/F smokers | 249 | 145 (40.2) | 104 (35.9) |  |
| Effusion |  |  |  |  |  |
| WBC (×10^9^/L) | < 0.72 | 173 | 140 (38.8) | 33 (11.4) | < 0.001 |
|  | ≥ 0.72 | 478 | 221 (61.2) | 257 (88.6) |  |
| neutrophil (×10^9^/L) | < 0.88 | 502 | 242 (67.0) | 260 (89.7) | < 0.001 |
|  | ≥ 0.88 | 149 | 119 (33.0) | 30 (10.3) |  |
| lymphocyte (×10^9^/L) | < 0.8 | 303 | 246 (68.1) | 57 (19.7) | < 0.001 |
|  | ≥ 0.8 | 348 | 115 (31.9) | 233 (80.3) |  |
| Total protein (g/L) | < 47.55 | 323 | 258 (71.5) | 65 (22.4) | < 0.001 |
|  | ≥ 47.55 | 328 | 103 (28.5) | 225 (77.6) |  |
| Glucose (mmol/L) | < 6.44 | 380 | 156 (43.2) | 224 (77.2) | < 0.001 |
|  | ≥ 6.44 | 271 | 205 (56.8) | 66 (22.8) |  |
| ADA (U/L) | < 22.75 | 312 | 280 (77.6) | 32 (11.0) | < 0.001 |
|  | ≥ 22.75 | 339 | 81 (22.4) | 258 (89.0) |  |
| LDH (U/L) | < 247.5 | 227 | 193 (53.5) | 34 (11.7) | < 0.001 |
|  | ≥ 247.5 | 424 | 168 (46.5) | 256 (88.3) |  |
| CA125 (U/ml) | < 1996.2  ≥ 1996.2 | 514  137 | 261 (72.3)  100 (27.7) | 253 (87.2)  37 (12.8) | <0.001 |
| CA19-9 (U/ml) | <2.54  ≥ 2.54 | 375  276 | 226 (62.6)  135 (37.4) | 149 (51.4)  141 (48.6) | 0.004 |
| Serum |  |  |  |  |  |
| WBC (×10^9^/L) | < 8.68  ≥ 8.68 | 475  176 | 224 (62.0)  137 (38.0) | 251 (86.6)  39 (13.4) | < 0.001 |
| neutrophil (×10^9^/L) | < 5.89 | 442 | 203 (56.2) | 239 (82.4) | < 0.001 |
|  | ≥ 5.89 | 209 | 158 (43.8) | 51 (17.6) |  |
| lymphocyte (×10^9^/L) | < 0.52  ≥ 0.52 | 73  578 | 52 (14.4)  309 (85.6) | 21 (7.2)  269 (92.8) | 0.004 |
| hsCRP (mg/L) | < 14.47 | 208 | 146 (40.4) | 62 (21.4) | < 0.001 |
|  | ≥ 14.47 | 443 | 215 (59.6) | 228 (78.6) |  |
| ESR (mm/h) | < 12.5 | 73 | 55 (15.2) | 18 (6.2) | < 0.001 |
|  | ≥ 12.5 | 578 | 306 (74.8) | 272 (93.8) |  |
| ADA (U/L) | < 8.05 | 154 | 101 (28.0) | 53 (18.3) | 0.004 |
|  | ≥ 8.05 | 497 | 260 (72.0) | 237 (81.7) |  |
| LDH (U/L) | < 158.5 | 153 | 97 (26.9) | 56 (19.3) | 0.026 |
|  | ≥ 158.5 | 498 | 264 (73.1) | 234 (80.7) |  |
| CA125 (U/ml) | < 58.95 | 245 | 170 (47.1) | 75 (25.9) | < 0.001 |
|  | ≥ 58.95 | 406 | 191 (52.9) | 215 (74.1) |  |
| CA19-9 (U/ml) | < 8.52 | 375 | 164 (45.4) | 201 (69.3) | < 0.001 |
|  | ≥ 8.52 | 276 | 187 (54.6) | 89 (30.7) |  |
| Ratio |  |  |  |  |  |
| CA125 ratio | < 12.43 | 364 | 159 (44.0) | 205 (70.7) | < 0.001 |
|  | ≥ 12.43 | 287 | 202 (56.0) | 85 (29.3) |  |
| CA19-9 ratio | < 0.33 | 250 | 206 (57.1) | 44 (15.2) | < 0.001 |
|  | ≥ 0.33 | 401 | 155 (42.9) | 246 (84.8) |  |
| Effusion LDH/ADA | < 17.07 | 309 | 85 (23.5) | 224 (77.2) | < 0.001 |
|  | ≥ 17.07 | 342 | 276 (76.5) | 66 (22.8) |  |
| Serum NLR | < 6.15 | 447 | 218 (60.4) | 229 (79.0) | < 0.001 |
|  | ≥ 6.15 | 204 | 143 (39.6) | 61 (21.0) |  |
| ADA/hsCRP | < 0.44 | 239 | 186 (51.5) | 53 (18.3) | <0.001 |
|  | ≥ 0.44 | 412 | 175 (49.5) | 237 (81.7) |  |

TB, tuberculous; TPE, tuberculous pleural effusion; WBC, white blood cell; ADA, adenosine deaminase; LDH, lactatedehy drogenase; CA125, carbohydrate antigen 125; CA19-9, carbohydrate antigen 19-9; hsCRP, high-sensitivity C-reactive protein; ESR, erythrocyte sedimentation rate; CA125 ratio, effusion/serum CA125; CA19-9 ratio, effusion/serum CA19-9; Effusion LDH/ADA, effusion LDH/ effusion ADA; Serum NLR, serum neutrophil/ serum lymphocyte; ADA/hsCRP, effusion ADA/ serum hsCRP

Chi-Square (X^2^) or Fisher Exact tests were used to compare the differences in categorical variables
