# Supplementary material for: Development and validation of a prediction model for tuberculous pleural effusion: a large cohort study and external validation
Source: Respir Res. 2022 May 27;23:134. doi: 10.1186/s12931-022-02051-4 (PMC9145463; doi:10.1186/s12931-022-02051-4)
Supplement: Supplementary file 2 — Additional file 2: Table S2. Univariate logistic regression analysis of the clinical characteristics in the training set. [file 12931_2022_2051_MOESM2_ESM.docx]

Table S2 Univariate logistic regression analysis of the clinical characteristics in the training set

| Variables |  | OR (95%CI) | *P* value | AUC |
| --- | --- | --- | --- | --- |
| Age (years) | < 54 | Reference |  |  |
|  | ≥ 54 | 0.199 (0.141-0.279) | < 0.001 | 0.725 |
| Effusion |  |  |  |  |
| WBC (×10^9^/L) | < 0.72 | Reference |  |  |
|  | ≥ 0.72 | 4.933 (3.242-7.507) | < 0.001 | 0.603 |
| Neutrophil (×10^9^/L) | < 0.88 | Reference |  |  |
|  | ≥ 0.88 | 0.236 (0.153-0.366) | < 0.001 | 0.591 |
| Lymphocyte (×10^9^/L) | < 0.8 | Reference |  |  |
|  | ≥ 0.8 | 9.192 (6.351-13.305) | < 0.001 | 0.782 |
| Total protein (g/L) | < 47.55 | Reference |  |  |
|  | ≥ 47.55 | 8.671 (6.060-12.406) | < 0.001 | 0.781 |
| Glucose (mmol/L) | < 6.44 | Reference |  |  |
|  | ≥ 6.44 | 0.224 (0.159-0.316) | < 0.001 | 0.639 |
| ADA (U/L) | < 22.75 | Reference |  |  |
|  | ≥ 22.75 | 27.870 (17.899-43.396) | < 0.001 | 0.860 |
| LDH (U/L) | < 247.5 | Reference |  |  |
|  | ≥ 247.5 | 8.650 (5.722-13.076) | < 0.001 | 0.638 |
| CA125 (U/ml) | < 1996.2 | Reference |  |  |
|  | ≥ 1996.2 | 0.382 (0.252-0.578) | < 0.001 | 0.562 |
| CA19-9 (U/ml) | < 2.54 | Reference |  |  |
|  | ≥ 2.54 | 1.584 (1.157-2.168) | 0.004 | 0.549 |
| Serum |  |  |  |  |
| WBC (×10^9^/L) | < 8.68 | Reference |  |  |
|  | ≥ 8.68 | 0.253 (0.170-0.377) | < 0.001 | 0.637 |
| Neutrophil (×10^9^/L) | < 5.89 | Reference |  |  |
|  | ≥ 5.89 | 0.275 (0.191-0.397) | < 0.001 | 0.634 |
| Lymphocyte (×10^9^/L) | < 0.52 | Reference |  |  |
|  | ≥ 0.52 | 2.156 (1.266-3.671) | 0.005 | 0.502 |
| hsCRP (mg/L) | < 14.47 | Reference |  |  |
|  | ≥ 14.47 | 2.497 (1.759-3.546) | < 0.001 | 0.538 |
| ESR (mm/h) | < 12.5 | Reference |  |  |
|  | ≥ 12.5 | 2.716 (1.557-4.739) | < 0.001 | 0.520 |
| ADA (U/L) | < 8.05 | Reference |  |  |
|  | ≥ 8.05 | 1.737 (1.192-2.530) | 0.004 | 0.547 |
| LDH (U/L) | < 158.5 | Reference |  |  |
|  | ≥ 158.5 | 1.535 (0.987-2.230) | 0.024 | 0.529 |
| CA125 (U/ml) | < 58.95 | Reference |  |  |
|  | ≥ 58.95 | 2.551 (1.826-3.565) | < 0.001 | 0.614 |
| CA19-9 (U/ml) | < 8.52 | Reference |  |  |
|  | ≥ 8.52 | 0.412 (0.298-0.570) | < 0.001 | 0.613 |
| Ratio |  |  |  |  |
| CA125 ratio | < 12.43 | Reference |  |  |
|  | ≥ 12.43 | 0.326 (0.235-0.453) | < 0.001 | 0.654 |
| CA19-9 ratio | < 0.33 | Reference |  |  |
|  | ≥ 0.33 | 7.430 (5.068-10.893) | < 0.001 | 0.674 |
| Effusion LDH/ADA | < 17.07 | Reference |  |  |
|  | ≥ 17.07 | 0.091 (0.063-0.131) | < 0.001 | 0.835 |
| Serum NLR | < 6.15 | Reference |  |  |
|  | ≥ 6.15 | 0.406 (0.282-0.578) | < 0.001 | 0.602 |
| ADA/hsCRP | < 0.44 | Reference |  |  |
|  | ≥ 0.44 | 4.753 (3.308-6.828) | < 0.001 | 0.679 |

TB, tuberculous; TPE, tuberculous pleural effusion; OR, odds ratio; AUC, area under the curve; WBC, white blood cell; ADA, adenosine deaminase; LDH, lactatedehy drogenase; CA125, carbohydrate antigen 125; CA19-9, carbohydrate antigen 19-9; hsCRP, high-sensitivity C-reactive protein; ESR, erythrocyte sedimentation rate; CA125 ratio, effusion/serum CA125; CA19-9 ratio, effusion/serum CA19-9; Effusion LDH/ADA, effusion LDH/ effusion ADA; Serum NLR, serum neutrophil/ serum lymphocyte; ADA/hsCRP, effusion ADA/ serum hsCRP
